# Supplementary material for: The causal relationship between the human gut microbiota and pyogenic arthritis: a Mendelian randomization study
Source: Front Cell Infect Microbiol. 2024 Nov 26;14:1452480. doi: 10.3389/fcimb.2024.1452480 (PMC11629706; doi:10.3389/fcimb.2024.1452480)
Supplement: Supplementary file 1 [file DataSheet1.zip › mendelian test/ebi-a-GCST90027562.csv_leave_one_out.pdf]

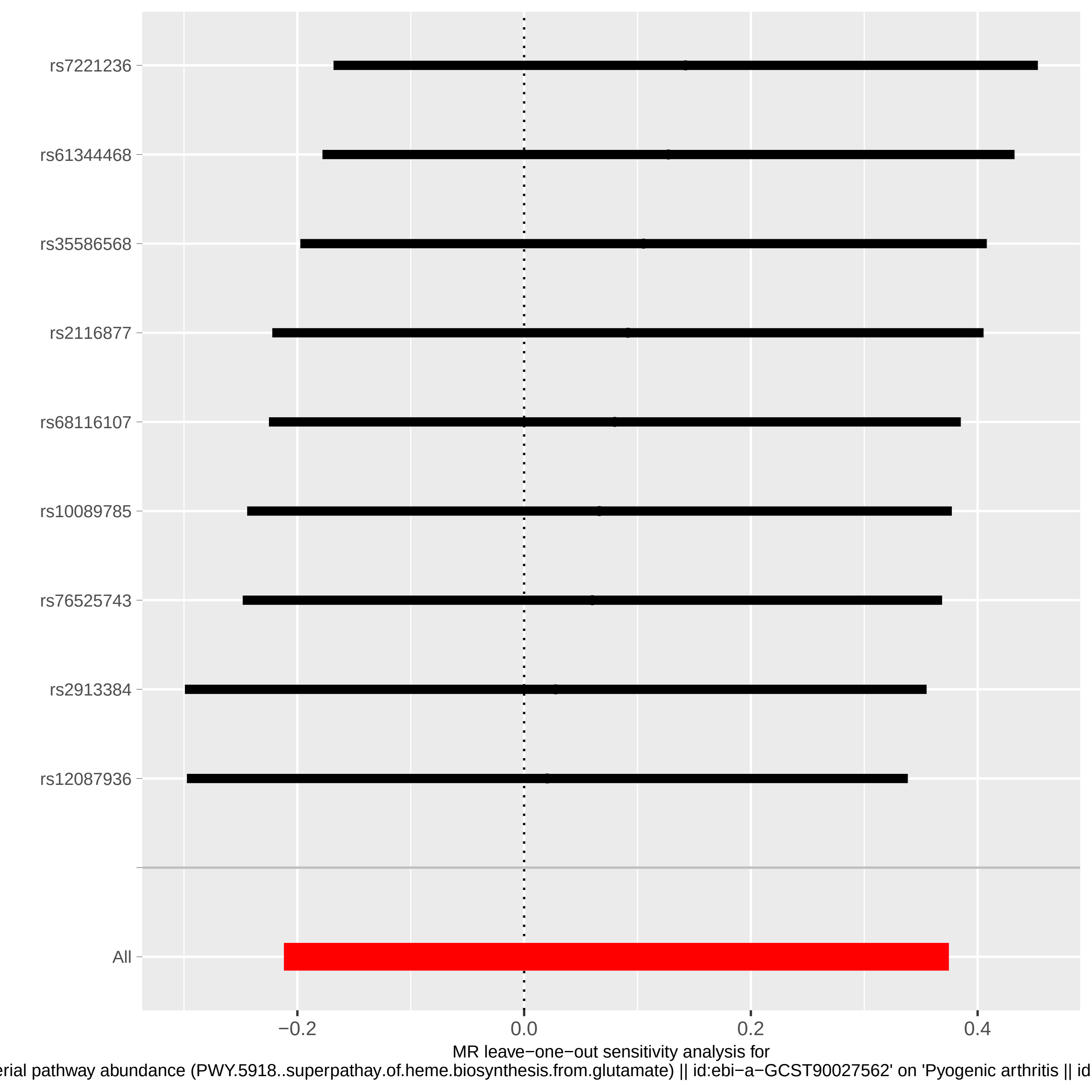

rs7221236
rs61344468
rs35586568
rs2116877
rs68116107
rs10089785
rs76525743
rs2913384
rs12087936
All
−0.2
0.0
0.2
0.4
MR leave−one−out sensitivity analysis for
erial pathway abundance (PWY.5918..superpathay.of.heme.biosynthesis.from.glutamate) || id:ebi−a−GCST90027562' on 'Pyogenic arthritis || id
